# Providing web-based mental health services to at-risk women

**DOI:** 10.1186/1472-6874-11-38

**Published:** 2011-08-19

**Authors:** Ellen L Lipman, Meghan Kenny, Elsa Marziali

**Affiliations:** 1 Department of Psychiatry and Behavioural Neurosciences, McMaster University, 1280 Main St. W., Hamilton, Ontario, Canada L8S 4K1; 2 Offord Centre for Child Studies, Patterson 206, Chedoke Site, 566 Sanatorium Road, Hamilton, Ontario, Canada. L9C 1Y3; 3 Baycrest, 3560 Bathurst Street, Toronto, Ontario, Canada M6A 2E1

**Keywords:** E-health, Community-based intervention, Lone Mothers, Group intervention

## Abstract

**Background:**

We examined the feasibility of providing web-based mental health services, including synchronous internet video conferencing of an evidence-based support/education group, to at-risk women, specifically poor lone mothers. The objectives of this study were to: (i) adapt a face-to-face support/education group intervention to a web-based format for lone mothers, and (ii) evaluate lone mothers' response to web-based services, including an online video conferencing group intervention program.

**Methods:**

Participating mothers were recruited through advertisements. To adapt the face-to-face intervention to a web-based format, we evaluated participant motivation through focus group/key informant interviews (n = 7), adapted the intervention training manual for a web-based environment and provided a computer training manual. To evaluate response to web-based services, we provided the intervention to two groups of lone mothers (n = 15). Pre-post quantitative evaluation of mood, self-esteem, social support and parenting was done. Post intervention follow up interviews explored responses to the group and to using technology to access a health service. Participants received $20 per occasion of data collection. Interviews were taped, transcribed and content analysis was used to code and interpret the data. Adherence to the intervention protocol was evaluated.

**Results:**

Mothers participating in this project experienced multiple difficulties, including financial and mood problems. We adapted the intervention training manual for use in a web-based group environment and ensured adherence to the intervention protocol based on viewing videoconferencing group sessions and discussion with the leaders. Participant responses to the group intervention included decreased isolation, and increased knowledge and confidence in themselves and their parenting; the responses closely matched those of mothers who obtained same service in face-to-face groups. Pre-and post-group quantitative evaluations did not show significant improvements on measures, although the study was not powered to detect these.

**Conclusions:**

We demonstrated that an evidence-based group intervention program for lone mothers developed and evaluated in face-to-face context transferred well to an online video conferencing format both in terms of group process and outcomes.

## Background

It is estimated that one in five Canadian adults will have a serious mental illness during their lifetime that merits treatment, but many may not receive this treatment [1,2]. Suboptimal care for mental health problems has been demonstrated for specific psychiatric disorders (e.g., depression [3]) and for specific populations including women and individuals in low-income households or neighbourhoods [3-6]. Barriers to service access and use include individual factors (e.g., costs of service or of transportation to service, childcare, attitude about seeking service) and contextual factors (e.g., organization of health services including location and timing of service) [7-12].

In Canada, 12.7% of Canadian families are led by lone mothers [13]. These mothers often have low levels of education and face economic stresses [14,15], social isolation, and mental health difficulties, characteristics associated with suboptimal mental health treatment. For example, lone mothers endorse higher levels of depressed mood and family stress as well as lower levels of social support compared with mothers from two-parent families [16-19].

E-health is a field that is developing at an extraordinary rate. The internet is a commonly used source of information related to mental health, with elevated use among those with a history of mental health problems [20].

Use of the internet to deliver mental health services has the potential to improve service access and to reduce waiting list delays. Internet-based mental health programs and services are available for a range of mental health problems, and have been evaluated for a variety of disorders and using different intervention formats [21-29]. Available web-based delivery formats for mental health programs include self-help websites, structured computer-administered therapy with or without computer-generated feedback, and therapies involving asynchronous and synchronous text-based contact with other users (internet support groups) or with therapists [25,26,28,30-32]. Only one study has examined groups provided with synchronous internet video conferencing, or real-time computer contact with video [33,34]. Marziali and colleagues successfully adapted a manualized face-to-face support group intervention to family caregivers of adults with dementia for delivery as an internet-based video conferencing group, including development of a secure web-site for video conferencing, and computer training for non-users [33,34]. Participants lived in remote areas, most were elderly and many had never used computers [33]. Almost all participants viewed the on-line group experience as positive and supportive [33,34].

We examined the feasibility of providing web-based mental health services, including synchronous internet video conferencing of an evidence-based support/education group [35], to at-risk women, specifically poor lone mothers, using the web-based platform developed by Marziali [36]. The objectives of this pilot project were to: (i) adapt a face-to-face support/education group intervention to a web-based format for lone mothers, and (ii) evaluate lone mothers' response to web-based services, including an online video conferencing group intervention program.

## Methods

### Participants

Mothers were recruited using flyers posted in the community and at medical offices asking about interest in a group for lone mothers. Participants responding were screened by telephone using questions about parenting on their own, having a child 3- to 9- years of age, and economic status. Eligibility criteria were: (1) at least one child 3-9 years of age, (2) spoke English, (3) no acute psychiatric crisis (e.g., suicidal behaviour) or threat of violence (e.g., by ex-partner), and (4) provided informed written consent to participate. Seven women participated in focus group and key informant interviews to evaluate participant motivation for engaging in web-based services. Subsequently, fifteen women participated in two pilot groups (n for Groups 1 and 2 = 8, 7 respectively). All provided informed signed consent prior to participation. This project was approved by the Research Ethics Board of Hamilton Health Sciences/Faculty of Health Sciences, McMaster University (REB #08-284).

### Intervention

The face-to-face support/education group intervention that was previously evaluated [35] was adapted to a web-based format. The intervention is aimed at poor lone mothers of 3-to 9-year old children, and delivered over 10 weeks, 1.5 hours per week. Each support group includes 6 to 10 women and two trained leaders. An intervention manual is used to guide structure, content and implementation of the program. Content of the mothers' group covers two main areas: child themes (e.g., normal and deviant development and behavior, behavior management, child welfare) and maternal themes (e.g., social isolation, financial stress, coping, relationships). There is no specified order in introducing content, as experience with similar groups [35,37,38] has shown that all content areas are covered over the course of the program. Leaders use group processes to create a safe and therapeutic group, use cognitive behavioral techniques and provide structured group counseling. We adapted the face-to-face intervention training manual for use in a web-based group environment. In addition, clinical consultations were held after each online group session and group videoconferencing sessions were viewed to insure adherence to the intervention protocol.

### Technology

A password-protected web site (Caring for Me, CFM ^©^) provided the platform for the delivery of the intervention. The website supports the videoconferencing intervention program and provided links to an internal e-mail (between group members and leaders), a message board, and a resource manual containing information on child development and behavior management, stress and financial management, and electronic and local resources relevant to children and their lone mothers. A computer training manual used to access the CFM^© ^website was adapted for use with the study participants. Equipment (computer, webcams and high speed internet access) was provided for the duration of the project to all participants who did not have this equipment. We provided computers for 93% (14/15) of group participants and paid for one year of high-speed internet for all participants. In addition, technical training on the basics of using computers to access the Internet and more specifically to negotiate the CFM^© ^website was provided for each group participant.

### Adaptation of face-to-face support/education group intervention to a web-based format

As part of the process of adapting the face-to-face support/education group intervention to a web-based format, we evaluated participant motivation for engaging in web-based services through one focus group and three key informant interviews, totaling 7 women. All mothers demonstrated a strong interest in participating in the online group program, exceeding participation rates in the previous RCT [35] and in other community-based trials for high-risk groups [39,40]. Preference was for a later evening group. In terms of computer experience, most (71%, 5/7) had computers and all said they had used the internet.

### Evaluation of lone mothers' response to web-based services, including an online video conferencing group intervention program

To evaluate participant response to web-based services, we ran two pilot groups (n for Groups 1 and 2 = 8, 7 respectively). The first pilot group ran for 8 weeks, beginning mid-July 2009 and the second group ran for 10 weeks, beginning November 2009. The first group was shortened from 10 to 8 sessions, with session cancellations due to technical difficulties (specifically changes to hospital and university server firewalls) and group leader illness. During the 8 group sessions that occurred, there were some technical difficulties (including in-home difficulties with volume controls and headsets) during the early group sessions. During these audio difficulties, the text box option allowed communication between group members (see next paragraph). For Group 2, seven women initially agreed to participate, but one did not follow through with any of the group sessions. Sessions were held at 8-9:30 p.m. on Wednesday evenings.

We utilized two types of videoconferencing software. For the first group, the software allowed up to 9 viewing streams plus the conference facilitator who passed the outgoing stream to the current speaker. The speaker (active window) was surrounded by video shots (not live) of the participants. This software included a text box option, where group members could communicate with each other and with the group leaders during the group sessions. For the second group, the software allowed 10 simultaneous active windows, and less image freezing and voice lag. Most technical difficulties were resolved through systematic problem solving (e.g., telephone consultation regarding settings on participants' home computers) and occasional consultation with personnel associated with Marziali.

### Data Collection

**Figure 1:**
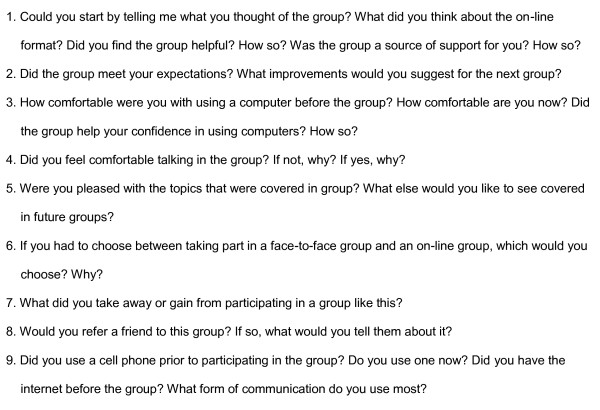
**Interview Guide (post-group)**.

Concurrent mixed methods were used. Quantitative data were collected at pre- and post-group from participants (see Measures section below). Qualitative assessments were done post-group using semi-structured interviews (interview guide shown in Figure 1). All assessment data were collected at home visits. Participants were given an honorarium on each occasion of data collection ($20).

For the analysis of group process and assessment of reliable adherence to intervention protocol, all video conferencing sessions from both groups were archived. Following termination of the intervention program all participants were interviewed to gather information about what participants thought about the group including the online format, comfort and confidence using the computer, and perceptions of helpfulness of the group intervention. All follow-up interviews lasted 1 to 2 hours, were audio recorded and transcribed verbatim. The interviewer also maintained field notes over the course of data collection. The interviewer was female, held a masters degree (M.A.) and had extensive experience conducting qualitative interviews.

### Descriptive Measures

Socio-demographic and health information included maternal age (years), number of children, child age, history of treatment for a mental health problem (any treatment for "nerves" or nervous condition ever, or in the last 6 months), current use of medication for a mental health problem (or trouble with nervous condition), education (highest grade/level completed among secondary school or less, secondary school, some post-secondary education, completed postsecondary education options), employment (worked at a job or business anytime in the past year), financial pressure (feels "money is a struggle", yes/no), and living in subsidized housing (yes/no).

Questionnaire data included several self-completed measures. Low mood was assessed using the Center for Epidemiological Studies Depression Scale [CES-D; Devins & Orme,1985; Radloff,1977][41,42], a 20-item self-report measure of psychological distress, including cognitive, affective and behavioural "state" of depression and respective frequencies. Scores range from 0-60, with higher scores indicating more severe symptomatology. Internal consistency = .84 -.90 [41,42]. The CES-D has been extensively validated [43]. Self-esteem was assessed by the Rosenberg Self-Esteem Scale [44], a 10-item self-report of self-esteem or psychological coping. Scores range from 10-40, with higher scores indicating higher global self- esteem. Internal consistency ratings range from .72 to .87 [45]. Social support was measured by the Social Provisions Scale [46], a 24-item self-report measure of perceived social support (6 subscales: attachment, social integration, reassurance of worth, reliable alliance, guidance, opportunity for nurturance and total). Total scores range from 24-96. Internal consistency = .65 to .76 [46]. We use the total score. Parenting was measured using the Parenting Stress Index-Short Form, a 36-item scale asking parents about parent-child (dysfunctional) interactions, parental distress, and difficult child (each 12 items), and a total score [47]. Ratings are made on a 5-point scale (1, strongly agree to 5, strongly disagree, total scale range, 12-180; higher scores reflect more dysfunction). This form was derived from and is highly correlated with a longer version. Internal consistency is 0.80, and 6-month test-retest reliability is 0.68. We use the total score.

### Data Analyses

Quantitative data were analyzed using SPSS version 15 [48]. Means and variances for selected descriptive variables were calculated. Paired t-tests were used to compare pre-post scores.

Qualitative analyses were conducted on the recorded interviews using a conventional content analysis approach. The main benefit of the conventional approach is that it allows the researcher to draw information directly from the participants' responses while refraining from applying any theoretical assumptions or predetermined inferences about the data [49]. A research assistant reviewed all of the transcripts to ensure the accuracy of the transcription. Analysis of the data commenced with examining the interview transcripts and the interviewer notes. Preliminary codes emerging from the data were identified, using the interview guides and the evaluation questions to keep the context of the data in mind. Following this brief overview, phrases were highlighted in the transcripts and viewed in light of the corresponding category, while all examples of a particular category were grouped together. Finally, all of the categories were listed and examined in terms of more broad and overarching themes.

## Results

Characteristics of group participants are shown in Table 1. Mothers ranged from 24 to 42 years of age (most in their thirties), had 1-4 children (age range 1 to 13 years), and identified difficulties for themselves (e.g., mental health problems such as depression, anxiety and substance use) and their children (e.g., behavior problems).

**Table 1:** Baseline characteristics of mothers participating in web-based pilot groups

Characteristic	Pilot Group 1 (n = 8) Mean (SD) [range]	Pilot Group 2 (n = 6) Mean (SD) [range]
Age, yr	34.9 (6.9) [24-42]	30.5 (3.0) [25-34]
	(n)	(n)
#Children/family		
1	(8)	(8)
2	(4)	(5)
3	(2)	(2)
4	(0)	(1)
Child age, range, yr	1-13	3-15
Maternal education		
Some secondary or less	(3)	(2)
Completed secondary	(0)	(1)
Some postsecondary	(2)	(1)
Completed postsecondary	(3)	(2)
Employed in past year	(3)	(1)
Financial pressure	(7)	(5)
Subsidized housing	(7)	(2)
Trouble with nerves ever	(5)	(1)
Trouble with nerves last 6 months	(4)	(4)
Medication for nerves	(3)	(3)
	Mean (SD)	Mean (SD)
Mood^a^	23.7 (4.3)	29.5 (13.4)
Self-esteem^a^	22.4 (4.3)	24.8 (5.3)
Social support^a^	54.1 (7.2)	53.0 (5.6)
Parenting stress-total^a^	111.4 (19.8)	108.4 (29.2)

^a^All outcomes scored to reflect poor functioning.

### Responses to Group Intervention

We present the main conceptual themes that emerged from the qualitative analysis of follow-up interviews with mothers, focusing on experiences of participants after participating in the online group intervention program.

#### Decreased Isolation

One of the main themes reflected the participants' decreased sense of isolation. All of the women associated this decrease with having the opportunity to connect with other people experiencing similar struggles.

"Well I was feeling very isolated and I think some of the other ones were feeling like that. So it was nice to know that there are other mothers and it really made me appreciate my life more, because I thought, oh my god, I'm a victim. I felt isolated and oh my god, hopeless, defeated, and then I go online with these guys and I go "wow, I don't have it bad as much as I think". And you know what, maybe it is manageable for me and I can do this and like I said with the resources, I feel strong where before I didn't feel that at all."

"I always felt like with [my son], it was like I am the only one going through this. Hearing [the other group members] going through the same kind of struggles - it was neat hearing that. Like, I'm not the only one going through this!"

In follow-up interviews, all participants shared that they had formed at least one friendship through their involvement in the group and that they had maintained contact once the group concluded.

"I'm walking away knowing that I'm not the only one out there. I actually walked away with a couple of friends."

"I made some friends. That was a new thing. I don't have a lot of female friends to talk to, especially ones with kids who have similar issues [to mine]. We all have children with similar issues. So it's always a good conversation for us."

"[The group members] were making me feel that I wasn't alone. We do call each other to see how we're doing and we still give each other support."

#### Increased Knowledge

A second theme reflected the participants' enhanced knowledge of parenting. All of the mothers reported feeling better equipped to parent their children, based on the resources that were shared with other group members. With the exception of two mothers, all of the families had children with some sort of diagnosis (e.g., attention deficit hyperactivity disorder, obsessive-compulsive disorder). All of these participants indicated that other group members were able to recommend useful resources for their children of which they were not aware. As one participant explained,

"But the resources I think was very important for everyone because it just seemed like everyone was at different levels of you know been there, done that, well you haven't been there, well you try that, so you try that and with this or whatever. Like that is going to change your whole path of life so...if I didn't have this group I wouldn't have ever known that."

#### Gained Confidence

Participants discussed their perceptions of having gained confidence in themselves for managing their life challenges.

"I have strength now, like emotional strength because I have the support and that helps me because before I felt defeated and I felt hopeless. And that is not a nice feeling to feel when you are raising a kid. And I have the resources and the confidence to know that I am on the right track now."

"I think I gained more self-confidence on what I know and what resources I know."

Three of the mothers shared that other group members had inspired them to be a better advocate for themselves and for their children. They described feeling intimidated by healthcare providers in the past and hesitation about voicing their opinions. One participant in particular shared the transformation she saw in her herself,

"The group taught me that you have to stand up for your child. If you're not happy with those diagnoses, then you need to take that to the next level and go to another doctor, and get a second opinion. And I've found that I've been doing that more."

Responses to Using Technology for Accessing a Mental Health Service

Analysis of the participants' reflections on using technology to participate in an online group intervention program yielded several consistent response themes.

#### Less Threatening

When asked to share their thoughts on the web-based group format, the response was overwhelmingly positive. One of the main themes that emerged was the sense that the web-based format was less threatening than a face-to-face format. As a result, all of the women felt that they were quickly able to establish a sense of trust in the group and were more inclined to open up.

"I think people opened up quicker online rather than face-to-face. You're not feeling like, "are they judging me?". There's a little bit of that but not nearly as much as dealing with someone in the same room."

"When you're in your own environment you feel more comfortable. You feel like you can open up more."

When participants were asked to explain why they found the web-based group less threatening they cited the safety and comfort of being able to participate from their own home, and the sense of anonymity created by participating on-line. As two women described,

"I felt more comfortable [in the online group]. I think that probably helped people to open up because you're not in a strange place. I was at home and I was familiar with everything around me. So I think I was a little bit more relaxed. At home I could be me. I don't think I had as big of a wall up."

"I prefer the online group because I don't have to worry about my anxiety or panic attacks or thinking that someone's looking at me the wrong way. Because usually what I do in those situations is I end up crying and embarrassing myself and then I have to leave and then I don't go back. I would totally avoid them at all cost."

Many of the women in the group had been diagnosed with depression or anxiety issues and as a result were wary of taking part in face-to-face groups outside of their home. Two of the women shared how they had refrained from participating in groups in the past due to their anxiety.

#### Convenience

Another appealing feature of the web-based format noted by all participants was the convenience of not having to arrange childcare and transportation in preparation for the group. Groups were held later in the evening after children were in bed so childcare was not an issue.

"You don't have to worry about getting a sitter, you don't have to worry about leaving the house. You don't have to worry about working the kids into the schedule. It's just, you're home, go on your computer and you're here."

"I prefer the online group. The security and comfort of the home, it also gives you a chance to focus on the group and know that your kids are right there and you don't have to worry about picking them up at the sitter after group, ruining the kid's routine because then it ruins mom's routine. For me, it was more of a relief because there wasn't all this extra work you have to do just to get yourself to group."

#### Computer Experience

All participants noted that their comfort using computers and overall experience with computers had increased. Mothers reported using the internet more frequently to find out about resources for themselves and for their children.

Group leaders were asked about their experience with working in a technology supported clinical environment compared to facilitating the face-to-face groups. They reported that the introduction of the new videoconferencing software during implementation of the second group enhanced the group experience; they stated it seemed just like a face-to-face group.

### Pre-post Responses to Questionnaires

Mean responses on all outcome measures showed improvement. Pre- to post- mean scores (with standard deviations) combined for both groups (n = 14) were: for self-esteem 23.4 (4.7) to 21.7 (4.7); for mood 26.2 (12.2) to 22.5 (12.3); for social support 53.6 (6.4) to 49.7 (8.9); and for total parenting stress 110.1 (23.3) to 101.2 (29.2) (all scales coded to reflect poor functioning so a decrease in score represents an improvement). Improvements were non-significant on all measures (all p > 0.10 on paired t-tests).

## Discussion

Based on widespread difficulties with access to mental health services and the potential of e-health, we examined the feasibility of providing web-based mental health services, including synchronous internet video conferencing of an evidence-based support/education group [35], to at-risk women, specifically poor lone mothers. The objectives of this pilot project were to: (i) adapt a face-to-face support/education group intervention to a web-based format for lone mothers, and (ii) evaluate lone mothers' response to web-based services, including an online video conferencing group intervention program.

In the process of adapting the group intervention to a web-based format, strong motivation for the web-based services was encountered in focus group and key informant interviews. We were able to adapt the intervention training manual for use in a web-based group environment and ensure adherence to the intervention protocol based on viewing group sessions and discussion with the leaders, and to provide training to participants for successful internet and CFM^© ^website access.

To evaluate participant response to web-based services, we ran two pilot groups. Our participant mothers were multiply disadvantaged, as are many lone mothers and as were mothers participating in the previous face-to-face groups [35]. Many of the participant mothers were not employed and experienced financial pressure necessitating assisted housing. Scores on the CES-D scale greater than 16 are considered indicative of probable clinical depression [42]and baseline levels indicated by mothers participating in our trial exceed this threshold.

Evaluation of web-based services was positive for both the quantitative and the qualitative sources. Qualitative analysis of the follow up interviews yielded themes that illustrate the participants' perceptions of the online intervention program. Their responses identified key issues that contributed to their psychosocial difficulties in being a lone, economically disadvantaged parent. Benefits of the group intervention included decreased isolation, increased knowledge and confidence in themselves as parents. These themes closely matched those that emerged from our analysis of follow up responses of mothers that participated in our face-to-face groups [50]. While it is possible that the provision of a free computer and free internet access for one year may have influenced participant response to report about the intervention in positive way, the finding that the themes closely matched those emerging from the face-to-face groups is reassuring.

As part of the pilot study we also scored the quantitative evaluation measures. While it is not an objective of pilot studies to demonstrate statistically significant changes in outcomes [51], demonstration of the trend for improvement (though non-significant) on all measures in this study is encouraging. It is notable that, despite improvement at follow-up evaluation, post-group mood scores remained above the threshold for probable clinical depression [mean(sd) pre: 26.2(12.2), post: 22.5(12.3)] [42].

Responses to using technology for accessing a mental health service were also positive. In post-group qualitative interviews, themes emerging included a) the perception that group members were more open more quickly, b) the convenience of accessing the group intervention from home, and c) increased computer skills and confidence. For example, one mother with anxiety felt she would not have been able to attend a face-to-face group but was happy to have had the opportunity to participate in the web-based group and felt it was useful.

The rapid development and potential of technology was apparent during this pilot study. New improved videoconferencing software for web-based videoconferencing became available mid-way through the project. The new software allowed participants to see all other group members simultaneously and offered other advantages that allowed the web-based experience to more closely approximate the face-to-face experience. It is likely that continued technological developments will increase the ease with which web-based videoconferencing interventions can be delivered.

Limitations of this work should be noted. This work is based on a small sample of mothers who responded to posters advertising the group and may not represent the views or experience of all lone mothers. The provision of a free computer and free internet for a year may have influenced participants to be positive about the intervention, though the themes emerging in the quantitative interviews were consistent with those emerging in face-to-face groups where no computer or internet support was provided. Pre-post improvements on outcome measures may have been related to regression to the mean. However, post-ratings remained elevated (e.g., the CES-D scale) [42]. Statistical power was inadequate to evaluate pre-post differences on quantitative measures.

## Conclusions

We established that it was feasible to provide web-based mental health services, including synchronous internet video conferencing that replicated an evidence-based support/education group [35], to at-risk women, specifically poor lone mothers. The project was successful in demonstrating that an evidence-based group intervention program for lone mothers developed and evaluated in face-to-face context transferred well to an online video conferencing format both in terms of group process and outcomes. Quantitative and qualitative evaluations of the group experience paralleled outcome responses to face-to-face groups. Of considerable importance was the fact that the participants were enthusiastic about using technology to access a mental health service; in fact several preferred the online intervention group to a face-to-face, clinic-based group intervention.

While successful in completing our pilot study objectives, there is additional work required to further evaluate these web-based services for persons with mental health problems. While web-based services can play an important role in making mental health services more accessible, what would be the response of lone mothers who live in remote areas and have no alternative choices for receiving mental health services? Would our evidence-based intervention yield the same results for those mothers as demonstrated in this feasibility, pilot study?

## List of Abbreviations

CFM ^©^: Caring for Me^©^.

## Competing interests

The authors declare that they have no competing interests.

## Authors' contributions

EL designed and conducted the study and drafted the manuscript. MK performed technical procedures, participant recruitment, data collection, data analysis and data interpretation and assisted with manuscript preparation and review. EM assisted with study conceptualization, technical procedures, on-going project oversight and manuscript review. All authors have read and approved the final manuscript.

## Pre-publication history

The pre-publication history for this paper can be accessed here:

http://www.biomedcentral.com/1472-6874/11/38/prepub
